# *tert*-Butyl 2-sulfanylidene-2,3-dihydro-1*H*-imidazole-1-carboxyl­ate

**DOI:** 10.1107/S1600536812027924

**Published:** 2012-06-27

**Authors:** Pei-Chi Lee, Yi-Cin Guo, Bor-Hunn Huang, Ming-Jen Chen

**Affiliations:** a Department of Applied Cosmetology and Graduate Institute of Cosmetic Science, Hungkuang University, Taichung 433, Taiwan; b Department of Chemistry, National Chung Hsing University, Taichung 402, Taiwan

## Abstract

In the title mol­ecule, C_8_H_12_N_2_O_2_S, the imidazole ring forms a dihedral angle of 5.9 (2)° with the mean plane of the carboxyl­ate group. In the crystal, mol­ecules are linked by pairs of N—H⋯S hydrogen bonds, forming inversion dimers.

## Related literature

The title compound is a mercaptoimidazole derivative. For applications of mercaptoimidazole derivatives in the treatment of hyperpigmentation, see: Kasraee (2002 ▶); Kasraee *et al.* (2005 ▶) and for inhibiting tyrosinase, see: Liao *et al.* (2012 ▶). For related structures containing inter­molecular N—H⋯S hydrogen bonds, see: Krepps *et al.* (2001 ▶).
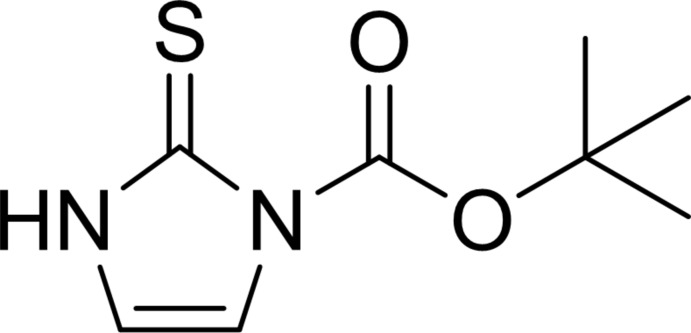


## Experimental

### 

#### Crystal data

C_8_H_12_N_2_O_2_S
*M* *_r_* = 200.26
Monoclinic, 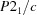

*a* = 6.8316 (3) Å
*b* = 8.8893 (5) Å
*c* = 17.5458 (15) Å
β = 90.789 (6)°
*V* = 1065.42 (12) Å^3^
*Z* = 4
Mo *K*α radiation
μ = 0.28 mm^−1^
*T* = 293 K
0.60 × 0.50 × 0.35 mm

#### Data collection

Agilent Xcalibur Sapphire3 Gemini diffractometer
Absorption correction: multi-scan (*CrysAlis PRO*; Agilent, 2011 ▶) *T*_min_ = 0.859, *T*_max_ = 1.000
4722 measured reflections
2472 independent reflections
1808 reflections with *I* > 2σ(*I*)
*R*_int_ = 0.047

#### Refinement

*R*[*F* ^2^ > 2σ(*F* ^2^)] = 0.070
*wR*(*F* ^2^) = 0.213
*S* = 1.09
2472 reflections
118 parameters
H-atom parameters constrained
Δρ_max_ = 0.37 e Å^−3^
Δρ_min_ = −0.67 e Å^−3^

### 

Data collection: *CrysAlis PRO* (Agilent, 2011 ▶); cell refinement: *CrysAlis PRO*; data reduction: *CrysAlis PRO*; program(s) used to solve structure: *SHELXS97* (Sheldrick, 2008 ▶); program(s) used to refine structure: *SHELXL97* (Sheldrick, 2008 ▶); molecular graphics: *SHELXTL* (Sheldrick, 2008 ▶); software used to prepare material for publication: *SHELXL97*.

## Supplementary Material

Crystal structure: contains datablock(s) I, global. DOI: 10.1107/S1600536812027924/lh5490sup1.cif

Structure factors: contains datablock(s) I. DOI: 10.1107/S1600536812027924/lh5490Isup2.hkl

Supplementary material file. DOI: 10.1107/S1600536812027924/lh5490Isup3.cml

Additional supplementary materials:  crystallographic information; 3D view; checkCIF report

## Figures and Tables

**Table 1:** Hydrogen-bond geometry (Å, °)

*D*—H⋯*A*	*D*—H	H⋯*A*	*D*⋯*A*	*D*—H⋯*A*
N1—H1*A*⋯S^i^	0.86	2.47	3.324 (2)	174

Symmetry code: (i) 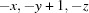.

**Fig. 1.**
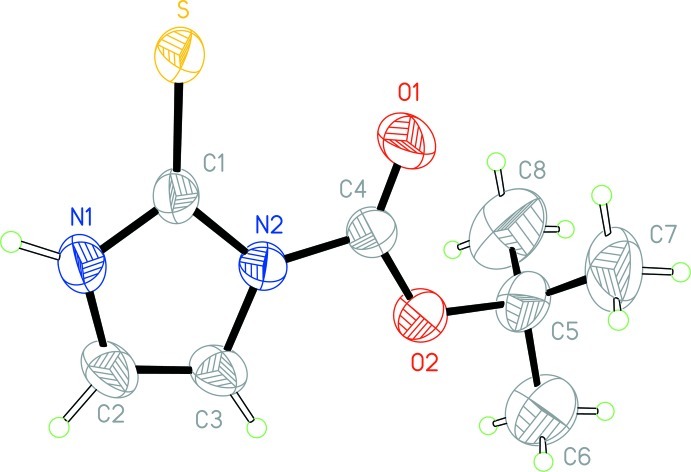
The molecular structure of (I), with ellipsoids for non-H atoms shown at the 50% probability level.

## supplementary crystallographic information

### Comment

1-methyl-2-mercaptoimidazole causes hypopigmentation by inhibiting tyrosinase in the clinical oral antithyroid medication (Kasraee (2002); Kasraee *et al.*, 2005). Ergothioneine has a significant effect on inhibiting tyrosinase enzyme activity, resulting from the presence of the sulfur substituent in the imidazole ring (Liao *et al.*, 2012). It shows that molecules with a 2-mercaptoimidazole group have potential as skin whitening agents. In this regard, we report here the synthesis and crystal structure of the title compound. The molecular structure of the title compound is shown in Fig. 1. The essentially planar imidazoline ring (C1/C2/C3/N1/N2) forms a dihedral angle of 5.9 (2)° with the mean plane of the carboxylate group (N2/C4/O1/O2). In the crystal, pairs of molecules are linked by N—H···S hydrogen bonds to form inversion dimers. Intermolecular N—H···S hydrogen bonds are highlighted in the literature by Krepps *et al.* (2001).

### Experimental

To a mixture of 2-mercaptoimidazole (351 mg, 3.5 mmole) and potassium carbonate (968 mg, 7 mmole) in 7 ml of *N*,*N*-dimethylformamide was added di-*tert*-butyl dicarbonate (1.1 ml, 5.2 mmol). The reaction mixture was stirred at 298 K for 24 h under N_2_ atmosphere. The resulting mixture was partitioned between ethyl acetate (40 ml) and H_2_O (20 ml). The organic layer was dried over MgSO_4_ and concentrated *in vacuo*. The residue was separated by chromatography over silica gel and eluted with hexane/ethyl acetate (3/7) to afford 297 mg of the title compound (I) in 42% yield. Single crystals suitable for X-ray measurements were obtained by recrystallization from a dichloromethane/hexane solution of the title compound at room temperature. Anal. Calcd for C_8_H_12_N_2_O_2_S: C, 47.98; H, 6.04; N, 13.99; Found: C, 47.86; H, 6.14; N, 13.92.

### Refinement

All H atoms were placed in geometrically idealized positions and treated as riding on their parent atoms, with C—H = 0.93 - 0.96 Å, N—H = 0.86 Å and *U*_iso_(H) > 1.2*U*_eq_(C,N) or 1.5 *U*_eq_(C_methyl_).

### Crystal data

**Table:**

C_8_H_12_N_2_O_2_S	-
*M**_r_* = 200.26	*D*_x_ = 1.248 Mg m^−^^3^
Monoclinic, *P*2_1_/*c*	Melting point: 439 K
Hall symbol: -P 2ybc	Mo *K*α radiation, λ = 0.71073 Å
*a* = 6.8316 (3) Å	Cell parameters from 1507 reflections
*b* = 8.8893 (5) Å	θ = 3.0–29.2°
*c* = 17.5458 (15) Å	µ = 0.28 mm^−^^1^
β = 90.789 (6)°	*T* = 293 K
*V* = 1065.42 (12) Å^3^	Parallelpiped, colourless
*Z* = 4	0.60 × 0.50 × 0.35 mm
*F*(000) = 424	

### Data collection

**Table:**

Agilent Xcalibur Sapphire3 Gemini diffractometer	2472 independent reflections
Radiation source: fine-focus sealed tube	1808 reflections with *I* > 2σ(*I*)
Graphite monochromator	*R*_int_ = 0.047
Detector resolution: 16.0690 pixels mm^-1^	θ_max_ = 29.2°, θ_min_ = 3.0°
ω scans	*h* = −9→9
Absorption correction: multi-scan (*CrysAlis PRO*; Agilent, 2011)	*k* = −11→11
*T*_min_ = 0.859, *T*_max_ = 1.000	*l* = −23→21
4722 measured reflections	

### Refinement

**Table:**

Refinement on *F*^2^	Primary atom site location: structure-invariant direct methods
Least-squares matrix: full	Secondary atom site location: difference Fourier map
*R*[*F*^2^ > 2σ(*F*^2^)] = 0.070	Hydrogen site location: inferred from neighbouring sites
*wR*(*F*^2^) = 0.213	H-atom parameters constrained
*S* = 1.09	*w* = 1/[σ^2^(*F*_o_^2^) + (0.120*P*)^2^] where *P* = (*F*_o_^2^ + 2*F*_c_^2^)/3
2472 reflections	(Δ/σ)_max_ < 0.001
118 parameters	Δρ_max_ = 0.37 e Å^−^^3^
0 restraints	Δρ_min_ = −0.67 e Å^−^^3^

### Special details

**Table:**

Experimental. Absorption correction: CrysAlisPro, Agilent Technologies, Version 1.171.35.19 (release 27-10-2011 CrysAlis171 .NET) (compiled Oct 27 2011,15:02:11) Empirical absorption correction using spherical harmonics, implemented in SCALE3 ABSPACK scaling algorithm.
Geometry. All e.s.d.'s (except the e.s.d. in the dihedral angle between two l.s. planes) are estimated using the full covariance matrix. The cell e.s.d.'s are taken into account individually in the estimation of e.s.d.'s in distances, angles and torsion angles; correlations between e.s.d.'s in cell parameters are only used when they are defined by crystal symmetry. An approximate (isotropic) treatment of cell e.s.d.'s is used for estimating e.s.d.'s involving l.s. planes.
Refinement. Refinement of *F*^2^ against ALL reflections. The weighted *R*-factor *wR* and goodness of fit *S* are based on *F*^2^, conventional *R*-factors *R* are based on *F*, with *F* set to zero for negative *F*^2^. The threshold expression of *F*^2^ > σ(*F*^2^) is used only for calculating *R*-factors(gt) *etc*. and is not relevant to the choice of reflections for refinement. *R*-factors based on *F*^2^ are statistically about twice as large as those based on *F*, and *R*- factors based on ALL data will be even larger.

### Fractional atomic coordinates and isotropic or equivalent isotropic displacement parameters (Å^2^)

**Table:**

	*x*	*y*	*z*	*U*_iso_*/*U*_eq_	
S	0.27124 (9)	0.62848 (7)	0.03744 (6)	0.0726 (4)	
O1	0.3738 (3)	0.9165 (2)	0.12448 (15)	0.0823 (8)	
O2	0.1365 (3)	1.08602 (18)	0.14513 (10)	0.0541 (5)	
N1	−0.1123 (3)	0.6966 (2)	0.03370 (11)	0.0468 (5)	
H1A	−0.1457	0.6126	0.0129	0.056*	
N2	0.0567 (3)	0.87696 (19)	0.08377 (11)	0.0389 (5)	
C1	0.0732 (3)	0.7337 (2)	0.05224 (13)	0.0415 (5)	
C2	−0.2421 (3)	0.8086 (3)	0.05181 (14)	0.0508 (6)	
H2A	−0.3770	0.8061	0.0440	0.061*	
C3	−0.1412 (3)	0.9201 (3)	0.08217 (13)	0.0472 (6)	
H3A	−0.1916	1.0109	0.0994	0.057*	
C4	0.2096 (3)	0.9595 (3)	0.11969 (14)	0.0469 (6)	
C5	0.2572 (4)	1.1899 (3)	0.19323 (15)	0.0587 (7)	
C6	0.1128 (6)	1.3169 (4)	0.2072 (2)	0.0962 (12)	
H6A	0.0046	1.2795	0.2360	0.144*	
H6B	0.0656	1.3553	0.1592	0.144*	
H6C	0.1769	1.3960	0.2352	0.144*	
C7	0.4278 (5)	1.2477 (4)	0.1471 (2)	0.0896 (11)	
H7A	0.5177	1.1669	0.1379	0.134*	
H7B	0.4935	1.3264	0.1748	0.134*	
H7C	0.3800	1.2865	0.0992	0.134*	
C8	0.3180 (8)	1.1119 (4)	0.2653 (2)	0.1084 (15)	
H8A	0.4083	1.0327	0.2538	0.163*	
H8B	0.2047	1.0702	0.2893	0.163*	
H8C	0.3798	1.1829	0.2992	0.163*	

### Atomic displacement parameters (Å^2^)

**Table:**

	*U*^11^	*U*^22^	*U*^33^	*U*^12^	*U*^13^	*U*^23^
S	0.0365 (4)	0.0442 (5)	0.1370 (8)	−0.0011 (3)	0.0015 (4)	−0.0349 (4)
O1	0.0419 (11)	0.0628 (12)	0.142 (2)	0.0071 (9)	−0.0166 (12)	−0.0510 (13)
O2	0.0475 (10)	0.0450 (9)	0.0696 (11)	0.0052 (8)	−0.0040 (8)	−0.0234 (8)
N1	0.0350 (10)	0.0458 (11)	0.0598 (11)	−0.0064 (9)	0.0021 (8)	−0.0122 (9)
N2	0.0304 (9)	0.0365 (9)	0.0501 (10)	0.0008 (7)	0.0051 (7)	−0.0070 (8)
C1	0.0348 (12)	0.0363 (11)	0.0535 (12)	−0.0065 (9)	0.0045 (9)	−0.0055 (10)
C2	0.0310 (11)	0.0640 (16)	0.0574 (14)	0.0018 (11)	0.0019 (10)	−0.0141 (12)
C3	0.0346 (12)	0.0546 (14)	0.0525 (13)	0.0079 (10)	0.0028 (9)	−0.0103 (11)
C4	0.0383 (12)	0.0404 (12)	0.0622 (14)	0.0022 (10)	0.0011 (10)	−0.0125 (11)
C5	0.0710 (18)	0.0431 (13)	0.0619 (15)	−0.0010 (13)	−0.0053 (13)	−0.0206 (12)
C6	0.104 (3)	0.0696 (19)	0.115 (3)	0.014 (2)	−0.003 (2)	−0.048 (2)
C7	0.090 (2)	0.0680 (19)	0.111 (3)	−0.0256 (19)	0.000 (2)	−0.029 (2)
C8	0.167 (5)	0.082 (3)	0.075 (2)	0.001 (2)	−0.038 (2)	−0.0115 (19)

### Geometric parameters (Å, º)

**Table:**

S—C1	1.668 (2)	C5—C8	1.496 (5)
O1—C4	1.186 (3)	C5—C7	1.518 (4)
O2—C4	1.312 (3)	C5—C6	1.521 (4)
O2—C5	1.492 (3)	C6—H6A	0.9600
N1—C1	1.345 (3)	C6—H6B	0.9600
N1—C2	1.374 (3)	C6—H6C	0.9600
N1—H1A	0.8600	C7—H7A	0.9600
N2—C1	1.394 (3)	C7—H7B	0.9600
N2—C3	1.405 (3)	C7—H7C	0.9600
N2—C4	1.417 (3)	C8—H8A	0.9600
C2—C3	1.316 (3)	C8—H8B	0.9600
C2—H2A	0.9300	C8—H8C	0.9600
C3—H3A	0.9300		
			
C4—O2—C5	120.9 (2)	O2—C5—C6	101.3 (2)
C1—N1—C2	112.04 (19)	C8—C5—C6	112.4 (3)
C1—N1—H1A	124.0	C7—C5—C6	109.8 (3)
C2—N1—H1A	124.0	C5—C6—H6A	109.5
C1—N2—C3	108.91 (18)	C5—C6—H6B	109.5
C1—N2—C4	125.90 (19)	H6A—C6—H6B	109.5
C3—N2—C4	124.82 (19)	C5—C6—H6C	109.5
N1—C1—N2	103.84 (18)	H6A—C6—H6C	109.5
N1—C1—S	126.03 (17)	H6B—C6—H6C	109.5
N2—C1—S	130.11 (16)	C5—C7—H7A	109.5
C3—C2—N1	107.6 (2)	C5—C7—H7B	109.5
C3—C2—H2A	126.2	H7A—C7—H7B	109.5
N1—C2—H2A	126.2	C5—C7—H7C	109.5
C2—C3—N2	107.6 (2)	H7A—C7—H7C	109.5
C2—C3—H3A	126.2	H7B—C7—H7C	109.5
N2—C3—H3A	126.2	C5—C8—H8A	109.5
O1—C4—O2	128.0 (2)	C5—C8—H8B	109.5
O1—C4—N2	123.7 (2)	H8A—C8—H8B	109.5
O2—C4—N2	108.25 (19)	C5—C8—H8C	109.5
O2—C5—C8	109.7 (2)	H8A—C8—H8C	109.5
O2—C5—C7	109.3 (2)	H8B—C8—H8C	109.5
C8—C5—C7	113.7 (3)		

### Hydrogen-bond geometry (Å, º)

**Table:**

*D*—H···*A*	*D*—H	H···*A*	*D*···*A*	*D*—H···*A*
N1—H1*A*···S^i^	0.86	2.47	3.324 (2)	174

Symmetry code: (i) −*x*, −*y*+1, −*z*.
